# Corrigendum: Genome-wide association study identifying variants related to performance and injury in high-performance athletes

**DOI:** 10.3389/ebm.2024.10348

**Published:** 2024-09-19

**Authors:** Jay R. Ebert, Agnes Magi, Eve Unt, Ele Prans, David J. Wood, Sulev Koks

**Affiliations:** ^1^ School of Human Sciences (Exercise and Sport Science), The University of Western Australia, Crawley, WA, Australia; ^2^ Department of Sports Medicine and Rehabilitation, Institute of Clinical Medicine, Faculty of Medicine, University of Tartu, Tartu, Estonia; ^3^ Sports Medicine and Rehabilitation Clinic, Tartu University Hospital, Tartu, Estonia; ^4^ Department of Anaesthesiology and Intensive Care, Tartu University Hospital, Tartu, Estonia; ^5^ School of Surgery, The University of Western Australia, Crawley, WA, Australia; ^6^ Perron Institute for Neurological and Translational Science, QEII Medical Centre, Nedlands, WA, Australia; ^7^ Centre for Molecular Medicine and Innovative Therapeutics, Murdoch University, Murdoch, Perth, WA, Australia

**Keywords:** genome-wide association, genetics, DNA, lower limb musculoskeletal injury

In the published article, there was an error in Figure 1 as published. While the Figure legend is correct, the image is incorrect. The corrected Figure 1 and its caption appear below.

**FIGURE 1:**
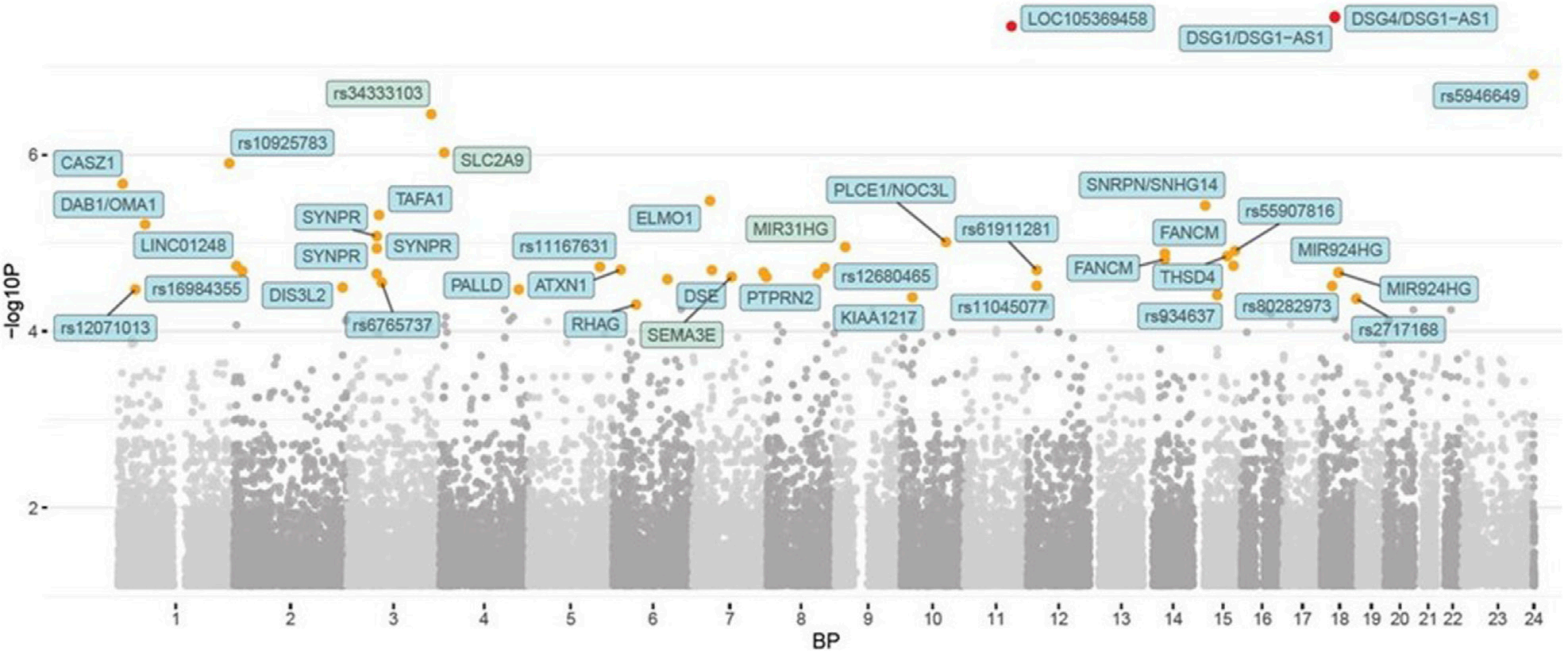
Manhattan plot representing the *p* values of the genome-wide association in reaching the podium (medalist) or not. The orange dots represent *p* < 10^−5^ while the red dots represent *p* < 10^−8^ (i.e., strong genome-wide significance).

In the published article, there was an error in Figure 2 as published. While the Figure legend is correct, the image is incorrect. The corrected Figure 2 and its appear below.

**FIGURE 2:**
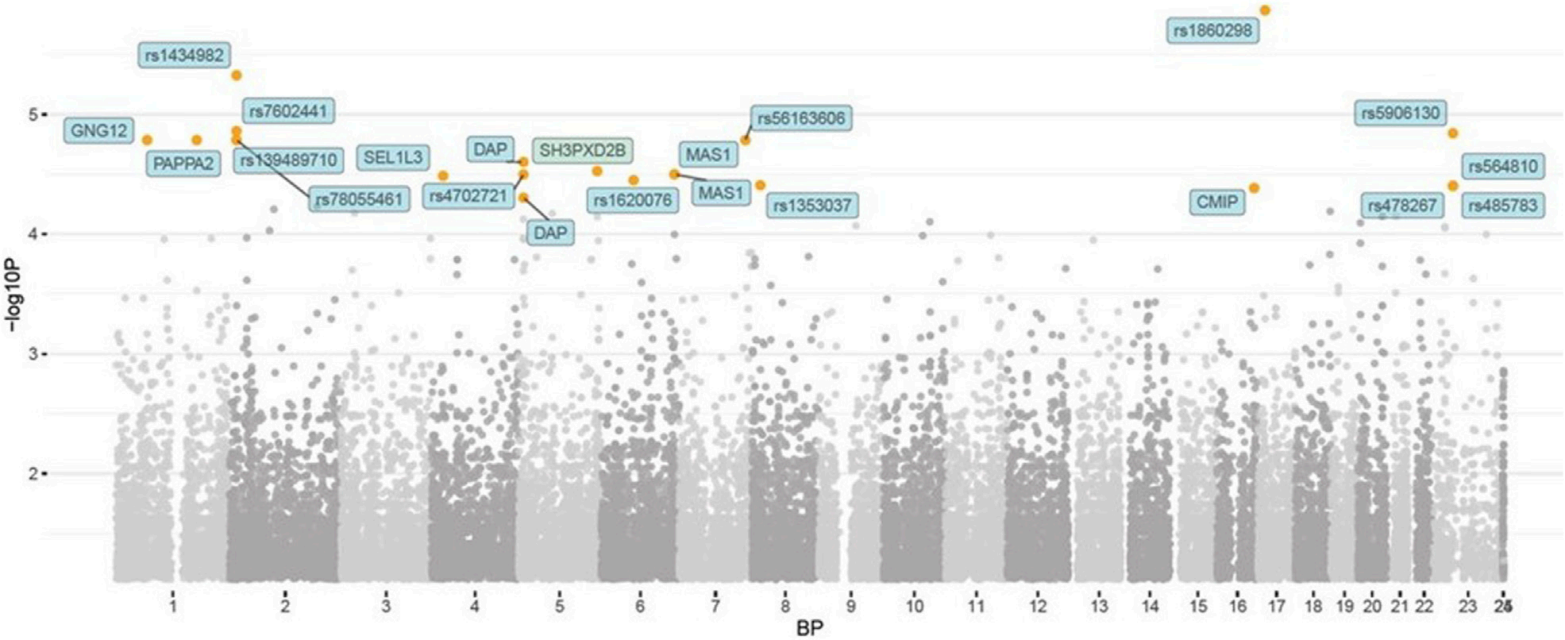
Manhattan plot representing the *p* values of the genome-wide association in being injured or not. The orange dots represent *p* < 10^−5^ while the red dots (N/A) represent *p* < 10^−8^ (i.e., strong genome-wide significance).

The authors apologize for these errors and state that this does not change the scientific conclusions of the article in any way.

